# eCAPRI: a novel tool combining clinical and imaging data for post-TAVI mortality prediction

**DOI:** 10.1007/s00330-025-12184-x

**Published:** 2025-12-20

**Authors:** Pierre-Jean Lartaud, Brahim Harbaoui, Angelo Della Corte, Aïssam Djahnine, Olivier Nempont, Jean-Michel Rouet, Anna Cecilia Vlachomitrou, Benjamin Riche, Muriel Rabilloud, Salim Si-Mohamed, Philippe Douek, François Derimay, Gilles Rioufol, Pierre Lantelme, Loïc Boussel

**Affiliations:** 1 Hospices Civils de Lyon, Lyon, France; 2 Philips Research France, Suresnes, France; 3 CREATIS UMR5220, INSERM U1044, INSA, Université de Lyon, Lyon, France

**Keywords:** TAVI, Risk assessment, Imaging biomarkers, Automated extraction, Aortic stenosis

## Abstract

**Objectives:**

Transcatheter aortic valve implantation (TAVI) is crucial for treating calcified aortic stenosis (CAS), yet post-procedural outcomes remain variable. The CAPRI score previously showed promising results in predicting 1-year all-cause mortality, by combining manually measured thoracic aortic calcium (TAC) volume with clinical risk factors. This study introduces an enhanced CAPRI score (eCAPRI), which automates TAC volume measurement and incorporates additional automatically extracted biomarkers from pre-operative CT scans.

**Materials and methods:**

TAC volume extraction was automated using a deep learning model trained on 66 patients and evaluated on 1111 CT scans. Additional automatically extracted imaging biomarkers were incorporated into the eCAPRI score, following the original methodology for biomarker selection. The eCAPRI score was trained on 765 pre-TAVI CT scans for one-year mortality prediction and then compared to CAPRI and EuroSCORE Logistic using AUC, bootstrap tests, and calibration curves on 192 CT scans.

**Results:**

Automated TAC segmentation achieved a mean Dice score of 0.777 ± 0.108. The eCAPRI score included body surface area (BSA)-indexed right ventricle volume, BSA-indexed pulmonary arteries max diameter, and abdominal muscles surface at L3 level in addition to automatically computed TAC volume and clinical biomarkers previously identified in the CAPRI score. On the evaluation dataset, eCAPRI showed an AUC of 0.731, outperforming CAPRI (AUC = 0.669) and EuroSCORE Logistic (AUC = 0.588) significantly (*p* = 0.034), with better calibration.

**Conclusion:**

The eCAPRI score, combining fully automated TAC volume extraction and additional imaging biomarkers, improved one-year mortality prediction over CAPRI and EuroSCORE Logistic. It may support standardized risk stratification in TAVI patients.

**Key Points:**

***Question*** *Can an eCAPRI using imaging biomarkers from pre-operative CT scans improve the prediction of one-year mortality in patients undergoing transcatheter aortic valve replacement*?

***Findings*** *The eCAPRI score, integrating automated TAC volume and additional biomarkers, outperformed CAPRI and EuroSCORE Logistic in predicting one-year mortality (AUC = 0.731, p = 0.034)*.

***Clinical relevance*** *The eCAPRI score provides a standardized approach to mortality risk assessment in transcatheter valve procedures. By improving prediction accuracy, it supports more informed clinical decisions and personalized care planning, ultimately contributing to better outcomes for patients undergoing TAVI*.

**Graphical Abstract:**

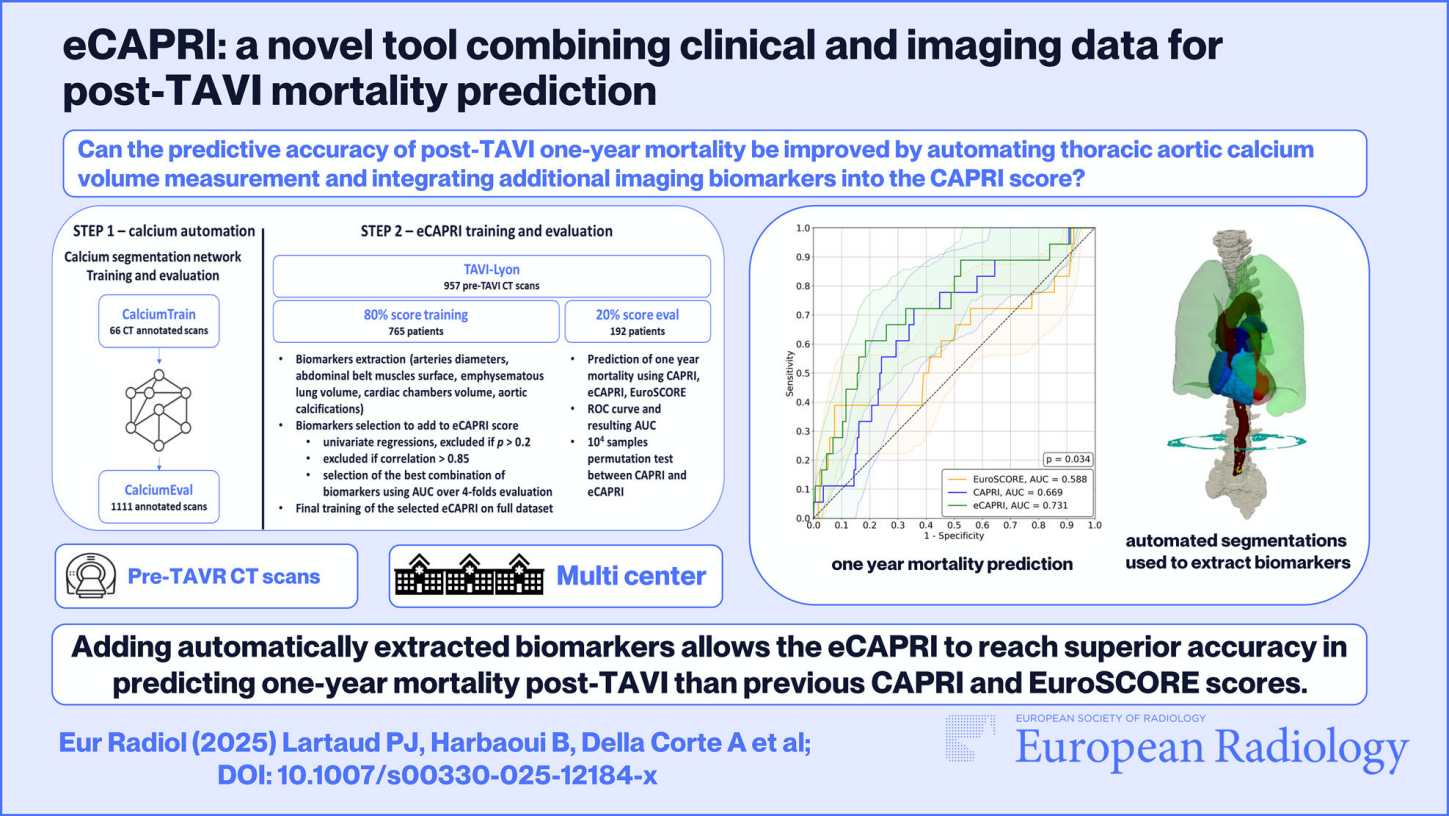

## Introduction

Over the last decade, transcatheter aortic valve implantation (TAVI) [1] has emerged as a validated alternative to surgical aortic valve replacement for the treatment of severe aortic stenosis in high- and intermediate-risk patients, based on evidence from high-quality randomized controlled trials [2–4], and is currently being investigated for low-risk patients [4, 5]. Nevertheless, despite high procedural success rates and low complication rates in contemporary cohorts [6], up to 25% of high-risk patients still experience poor outcomes at one-year follow-up [7], largely due to their significant burden of comorbidities.

Therefore, many survival studies [8–13] aimed at developing outcomes prediction model after TAVI, using various regression algorithms (logistic regression, multilayer perceptron, gradient boost algorithm) fed with variables such as biological data (albumin, hemoglobin, creatinine, hematocrit), risk scores (STS-PROM, EuroSCORE II), demographics (use of beta blockers, body-mass index, age) and cardiovascular measurements (QRS duration, aortic valve peak gradient, LVEF, valves area, stroke volume). Among these studies, Lantelme et al [14] developed the CAPRI score, which includes thoracic aortic calcium (TAC) volume extracted from CT. TAC is correlated with a significant increase in the risk of cardiovascular [15, 16] and all-cause death [17, 18]. Lantelme et al [14] demonstrated that adding TAC volume to other clinical, biological, and demographic risk factors significantly improved the prediction of 1-year cardiovascular and all-cause mortality after TAVI with an AUC of 0.68. However, the manual annotation of TAC on CT scan is a time-consuming task, and is a major barrier to clinical adoption of the CAPRI score.

The interest in imaging biomarkers for long-term outcome prediction was recently further highlighted by the work of Pickhard et al [11, 12]. In a set of 9223 asymptomatic adult patients who underwent non-contrast CT scans for routine colorectal cancer screening, they integrated L3 muscle density, Agatston calcium score of the abdominal aorta, L1 trabecular bone density, liver density, visceral and subcutaneous fat ratio to the Framingham risk score, and reached an AUC of 0.811 for the prediction of 2-year mortality.

This study aims to develop an improved and automated CAPRI scoring system for the prediction of 1-year mortality after TAVI, using a deep learning model for TAC segmentation and the extraction of additional imaging biomarkers of clinical interest.

## Materials and methods

### Previous work—the CAPRI score

This work is built upon the original CAPRI score by Lantelme et al [14]. In the proposed scoring system, variable selection was performed through univariate Cox regressions. A likelihood ratio test [19] was then calculated for each regression to test whether a significant difference in risk of all-cause death can be attributed to the variable. The correlation between variables was then evaluated using Spearman, tetrachoric, or polychoric correlation coefficients (depending on the nature of the variables). Finally, the CAPRI score is computed using the selected variables as:

$${{CAPRI}}_{{pat}}={\sum }_{{X}_{{{\rm{o}}}}}^{{X}_{i}}{\beta }_{i}({X}_{i}\left({pat}\right)-{\bar{X}}_{i})$$

with *CAPRI*_*pat*_, the CAPRI score for a given patient, *X*_*i*_ the ith variable of interest (detailed in Table 1), and *β*_*i*_ the coefficient of the multivariate Cox regression associated with it.

**Table 1:** CAPRI score variables

Type	Data	Definition
Discrete	Male	Is the patient male (1) or female (0).
	PAPs available	Binary data indicating if PAPs is available (1) or not (0).
	Peripheral arterial disease	Diagnosis of peripheral arterial disease (1) or not (0).
	Respiratory failure	Diagnosis of respiratory failure (1) or not (0).
	TIA, stroke	Diagnosis of TIA and/or stroke (1) or not (0).
	Coronary disease	Diagnosis of coronary disease (1) or not (0).
	NYHA (34)	New York Heart Association heart failure score, from I (none) to IV (acute)
	Mitral regurgitation (35)	Mitral regurgitation score from I ( < 30 mL) to V ( > 75 mL)
		According to the intensity of blood reflux through the mitral valve.
	Clearance (36)	Kidney failure score, from I (clearance > 90, normal)
		to V (clearance < 15, terminal failure).
Continuous	Age (years)	Patient’s age when admitted for surgery.
	PAPs (x10 mmHg)	PAPs / 10. If not available, treated as zero.
	Aortic gradient (mmHg)	LV pressure gradient, allows the detection of aortic stenosis.
	Ejection fraction (\%)	The percentage of blood volume before and after LV contraction,
		used to characterize heart failure.
	TAC volume (mL)	TAC volume above L1, here computed using
		The automated calcium segmentation model.

*PAPs* pulmonary artery pressure, *TIA* transient ischemic attack, *NYHA* New-York Heart Association, *LV* left ventricle, *TAC* thoracic aorta calcium

### Automated aortic calcium scoring

#### Data

To train and evaluate the calcification segmentation network, we collected two retrospective datasets. The *CalciumTrain* set consists of 66 patients who underwent chest-abdomen-pelvis scans (FOV: 386.34 ± 47.77), including 43 unenhanced scans and 23 contrasted scans in the arterial phase. Calcifications were annotated using adaptive thresholding. Candidates were selected by thresholding at 130 HU in the aorta and extended to cover the entire calcification using a region-growing algorithm. Candidates were then validated manually.

The *CalciumEval* evaluation set consists of 1111 patients who underwent a pre-TAVI contrasted CT scan (arterial phase), with fields of view covering the heart or full chest (FOV: 232.25 ± 51.98 mm). Calcifications were manually annotated slice by slice (3D-Slicer version 4.10.2) by senior radiologists.

#### Implementation of the calcium segmentation network

We trained an end-to-end segmentation network (TensorFlow 2.10) using a masked binary cross-entropy cost function that penalizes candidates according to their location, thanks to heart (left and right atria and ventricles, myocardium, aorta, pulmonary arteries) and spine segmentation [20, 21], which have been applied to the *CalciumTrain* and *CalciumEval* datasets. The cost function can be written as:

$${MBCE}=\,\frac{1}{n}{\sum }_{i=1}^{n}\left(\right.\left({{{\rm{{\Upsilon }}}}}_{i}\cdot \log \left({\hat{{{\rm{Y}}}}}_{i}\right)+\left(1-{{{\rm{{\Upsilon }}}}}_{i}\right)\cdot \log \left(1-{\hat{{{\rm{Y}}}}}_{i}\right)\times {{SpiE}}_{i}\times {{VaE}}_{i}\times {{Ao}}_{i}\,\right)$$

Where *MBCE* is the masked binary cross entropy, *n* is the number of voxels, ${{{\rm{{\Upsilon }}}}}_{i}$ is the ground truth for the *i*th voxel, *SpiE*_*i*_*, VaE*_*i*_ are exclusion masks for the spine and the aortic and mitral valves, respectively, and *Ao*_*i*_ a dilated aorta segmentation. *Ao*_*i*_ dilation is performed using a 2/*sp* kernel size, with *sp* the spacing in mm. The spine mask *SpiE*_*i*_ is an inverted vertebrae bodies segmentation (1 − *seg*) dilated by a kernel of 1/*sp* size. Finally, *VaE*_*i*_ is calculated from distance maps of the heart chamber segmentations. Hence, the network needs only to learn the visual characteristics of the calcium without its localization, as calcium outside of the aorta are excluded through the masks. Therefore, using a smaller network is possible while using full image resolution.

As Gogin et al [22], we used a 3D U-Net architecture for the network, along with an Adam optimizer and a decreasing learning rate (0.88 factor after each epoch) starting from 10^−3^, with values initialized from a default uniform distribution. Before training, the images and segmentations of the *CalciumTrain* data set were cropped around the aorta according to the segmentation provided by the cardiovascular segmentation network, for GPU memory optimization reasons. The images nevertheless retain their full resolution.

To highlight calcium and limit the range of gray levels, the images are windowed between [−200, 1000] HU, then normalized between [−1, 1] before being supplied to the network. During training, the network takes as input a batch composed of 5 randomly chosen patches of dimensions [100, 100, 100] voxels, with corresponding ground truths and masks. 10% of the training set is used as a test set during training. The final model is chosen when the cost function has reached convergence.

#### Calcium segmentation evaluation

During evaluation, the same pre-processing is performed on scans of the *CalciumEval* set, and the masks are provided to the network alongside the images. The quality of the predictions is evaluated on the *CalciumEval* database by calculating the Dice coefficient between each raw prediction and the corresponding ground truth. Calcification volumes were also studied using a scatter plot and a Bland–Altman plot. The specificity and sensitivity are provided.

### The enhanced CAPRI score (eCAPRI) (CAS) score

#### Data

The *TAVI-Lyon* retrospective dataset [23], includes 957 patients (479 men, 478 women) who underwent TAVI between 2013 and 2021. The average age of the whole group is 82.35 ± 7.53, ranging from 33 to 98 years. Before TAVI, all patients underwent a gated thorax-abdomen-pelvis CT scan, after intravenous injection of iodinated contrast agent (60–90 mL at ~3–5 mL/s injection rate) in arterial or late arterial phase. Slice thickness was set under 1 mm, and tube voltage to either 100 (300 patients), 120 (650), or 140 kVp (8) adjusted to patient size. Images were obtained from 4 hospitals, and from different scanner models: Revolution GSI, Revolution CT, Revolution HD, Revolution EVO, LightSpeed VCT from GE Healthcare, Somatom Definition Flash, Somatom Definition AS+, Biograph64 from Siemens Healthineers AG, and iCT 256, Brilliance 64, Brilliance 40, Ingenuity CT, iQon Spectral CT from Koninklijke Philips N.V. TAVI was performed through different routes (femoral, subclavian, transapical). All patients were then followed for up to 1 year, either through phone or in-person checkups. Adverse events were confirmed through the national death registry, *France-Décès*. Of the 957 patients, 81 (9.30%) died during the study. None left the study before the end of the one-year follow-up. The *TAVI-Lyon*, *CalciumEval,* and *CalciumTrain* datasets do not share any common patients.

The set was randomly split at a ratio of 80/20% (765 and 192 patients, respectively) for training and evaluation of the algorithm, ensuring an equivalent proportion of patients with an adverse event. Statistics for imaging parameters, patient characteristics, co-morbidities, and other parameters for both sets are summarized in Table 2. Regarding comorbidities, high-risk features such as impaired renal function (Stage III or worse in over 50% of patients), NYHA Class III/IV heart failure (49%), and atrial fibrillation (31%) were frequent. The prevalence of comorbidities such as coronary artery disease (32%) and peripheral arterial disease (13%) was also substantial.

**Table 2:** All parameters of the *TAVI-Lyon* dataset, divided into a training and a validation dataset

Data		Dataset	
Type	Name	Train	Evaluation
CAPRI score	Events	No: 694, yes: 71	No: 174, yes: 18
	Age (years)	82.19 ± 7.59 [33.00, 98.00]	82.99 ± 7.27 [52.00, 97.00]
	PAPs (×10 mmHg)	3.10 ± 2.17 [0.00, 9.10]	3.28 ± 2.11 [0.00, 8.00]
	Aortic gradient (mmHg)	48.62 ± 15.71 [2.00, 119.00]	46.93 ± 14.73 [3.94, 106.00]
	LV ejection fraction (%)	56.70 ± 12.40 [14.00, 85.00]	57.65 ± 11.50 [22.00, 83.00]
	Male	No: 376, yes: 389	No: 102, yes: 90
	Clearance	Grade I: 39, grade II: 280, grade III: 352, grade IV: 79, grade V: 15	Grade I: 16, grade II: 53, grade III: 99, grade IV: 24
	PAPs available	No: 196, yes: 569	No: 44, yes: 148
	Peripheral arterial disease	No: 672, yes: 93	No: 163, yes: 29
	Respiratory failure	No: 612, yes: 153	No: 149, yes: 43
	TIA, stroke	No: 682, yes: 83	No: 175, yes: 17
	Coronary disease	No: 523, yes: 242	No: 119, yes: 73
	NYHA	Grade I: 143, grade II: 223, grade III: 340, grade IV: 59	Grade I: 35, grade II: 57, grade III: 89, grade IV: 11
	Mitral regurgitation	Grade I: 292, grade II: 326, grade III: 137, grade IV: 8, grade V: 2	Grade I: 78, grade II: 81, grade III: 29, grade IV: 3, grade V: 1
	TAC volume (mL)	3.79 ± 3.77 [0.00, 25.32]	4.28 ± 4.22 [0.03, 24.94]
Biomarkers extracted from CT imaging	Lung volume (mL)	1581.25 ± 585.08 [304.04, 4188.19]	1594.54 ± 590.57 [700.40, 3669.73]
	Emphysema volume (mL)	15.37 ± 50.68 [0.00, 1045.08]	11.74 ± 24.37 [0.00, 171.25]
	LA volume (mL)	122.72 ± 47.56 [31.57, 403.50]	123.05 ± 47.69 [41.03, 357.28]
	LV volume (mL)	124.93 ± 54.14 [30.55, 378.35]	117.93 ± 48.18 [40.83, 265.31]
	Myo volume (mL)	175.40 ± 47.39 [66.67, 394.35]	167.68 ± 49.96 [57.89, 383.97]
	RA volume (mL)	107.85 ± 58.90 [12.33, 434.49]	112.23 ± 64.43 [32.92, 374.33]
	RV volume (mL)	134.85 ± 54.56 [20.87, 449.21]	132.42 ± 50.49 [53.73, 310.66]
	Ao max radius (cm)	3.64 ± 1.46 [0.00, 8.16]	3.75 ± 1.34 [0.02, 7.21]
	PA max radius (cm)	1.68 ± 1.04 [0.01, 5.55]	1.68 ± 0.97 [0.02, 4.87]
	L3 level muscles surface (cm²)	120.32 ± 28.78 [2.44, 222.03]	117.81 ± 28.27 [63.02, 240.42]
	BSAi lungs volume (mL/m²)	873.23 ± 310.35 [212.58, 2168.32]	876.43 ± 312.65 [333.55, 1994.25]
	BSAi emphysema volume (mL/m²)	8.54 ± 27.45 [0.00, 541.06]	6.56 ± 13.58 [0.00, 89.27]
	BSAi LA volume (mL/m²)	67.83 ± 25.65 [20.05, 241.90]	67.35 ± 23.98 [20.66, 184.87]
	BSAi LV volume (mL/m²)	68.95 ± 28.49 [15.72, 195.63]	64.43 ± 24.62 [20.29, 135.29]
	BSAi Myo volume (mL/m²)	96.65 ± 23.96 [44.91, 232.17]	91.10 ± 22.25 [31.78, 184.31]
	BSAi RA volume (mL/m²)	59.33 ± 31.37 [7.46, 243.87]	61.31 ± 33.62 [16.58, 194.85]
	BSAi RV volume (mL/m²)	74.02 ± 27.70 [11.59, 223.78]	72.41 ± 26.17 [32.12, 167.80]
	BSAi Ao max radius (cm/m²)	2.02 ± 0.82 [0.00, 4.77]	2.08 ± 0.75 [0.01, 3.95]
	BSAi PA max radius (cm/m²)	0.95 ± 0.62 [0.00, 3.93]	0.94 ± 0.56 [0.01, 2.96]
	BSAi L3 level muscles surface (cm²/m²)	66.02 ± 12.26 [1.26, 101.29]	64.17 ± 11.76 [38.06, 100.72]
	Emphysema ratio (%)	0.72 ± 1.73 [0.00, 24.95]	0.59 ± 1.04 [0.00, 6.68]
CT parameters	KVP (kV)	100 kV: 244, 120 kV: 514, 140 kV: 7	100 kV: 56, 120 kV: 136
	Intensity (mA)	288.47 ± 107.60 [69.00, 1555.00]	282.91 ± 98.71 [81.00, 599.00]
	Exposure time (s)	1299.29 ± 478.64 [182.00, 2801.00]	1331.48 ± 471.24 [231.00, 2801.00]
	Interslice (mm)	0.53 ± 0.20 [0.39, 2.01]	0.52 ± 0.13 [0.40, 1.00]
	Spacing (mm)	0.78 ± 0.09 [0.46, 0.98]	0.78 ± 0.09 [0.52, 0.98]
	Slices number	1332.78 ± 312.00 [294.00, 2038.00]	1324.16 ± 280.92 [591.00, 1985.00]
	FOV (mm)	398.82 ± 46.77 [238.00, 500.00]	400.18 ± 47.19 [264.00, 500.00]
Demographic data, history, and comorbidities	BMI (kg/m²)	27.66 ± 19.87 [14.17, 402.83]	29.11 ± 34.04 [15.58, 494.26]
	BSA (m²)	1.82 ± 0.23 [1.20, 2.74]	1.83 ± 0.23 [1.23, 2.46]
	EuroSCORE	14.67 ± 11.49 [0.55, 81.00]	14.31 ± 11.32 [1.22, 74.80]
	Coronary bypass	No: 703, yes: 62	No: 176, yes: 16
	Coronary angioplasty	No: 572, yes: 193	No: 137, yes: 55
	Pacemaker	No: 654, yes: 111	No: 171, yes: 21
	Diabetes	No: 572, yes: 193	No: 141, yes: 51
	Atrial fibrillation	No: 536, yes: 229	No: 127, yes: 65
	Supraventricular rhythm disorder	No: 591, yes: 174	No: 143, yes: 49
	History of cardiac surgery	No: 729, yes: 36	No: 185, yes: 7
	≥ 2 APO in current year	No: 715, yes: 50	No: 178, yes: 14
	Dyspnea	No: 99, yes: 666	No: 28, yes: 164
	Anticoagulant treatment	No: 528, yes: 237	No: 124, yes: 68
	Reduced mobility	No: 702, yes: 63	No: 176, yes: 16
	History of infarction	No: 753, yes: 12	No: 189, yes: 3
	Renal insufficiency	Grade I: 485, grade II: 216, grade III: 46, grade IV: 18	Grade I: 129, grade II: 47, grade III: 14, grade IV: 2
	Pulmonary hypertension	Grade I: 465, grade II: 244, grade III: 56	Grade I: 109, grade II: 72, grade III: 11
	Aortic regurgitation	Grade I: 342, grade II: 307, grade III: 100, grade IV: 10, grade V: 6	Grade I: 80, grade II: 76, grade III: 30, grade IV: 2, grade V: 4

*TAC* thoracic aorta calcium, *LA* left atrium, *LV* left ventricle, *Myo* left ventricle myocardium, *RA* right atrium, *RV* right ventricle, *Ao* aorta, *PA* pulmonary arteries, *BSAi* BSA-indexed, *PAPs* pulmonary arteries pressure, *BMI* body-mass index, *APE* acute pulmonary edema, *TIA* transient ischemic attack, *BSA* body surface area

#### Biomarkers extraction

We aimed to include additional imaging biomarkers of clinical interest described in the literature [11, 12, 24, 25]. Among them, visceral-to-subcutaneous fat ratio at L1 level and density measurements (liver, abdominal belt muscles, vertebrae) were ruled out, as their values are biased by the presence of contrast agent. The following biomarkers were therefore investigated:

- The volumes of the cardiac chambers and myocardium in the diastolic phase (75%) have been proven in the literature to be correlated with post-TAVI mortality [8, 10].
- Maximum diameters of the aorta and pulmonary arteries, for the same reasons.
- The volumes of emphysematous and healthy lung tissues, along with their ratio, as chronic obstructive pulmonary disease (COPD) negatively impacted both short-term and long-term all-cause and cardiovascular survival [26].
- The surface area of the abdominal belt muscles at the L3 level, which are markers of sarcopenia, and overall bad patient health [25, 27, 28].

**Fig. 1:**
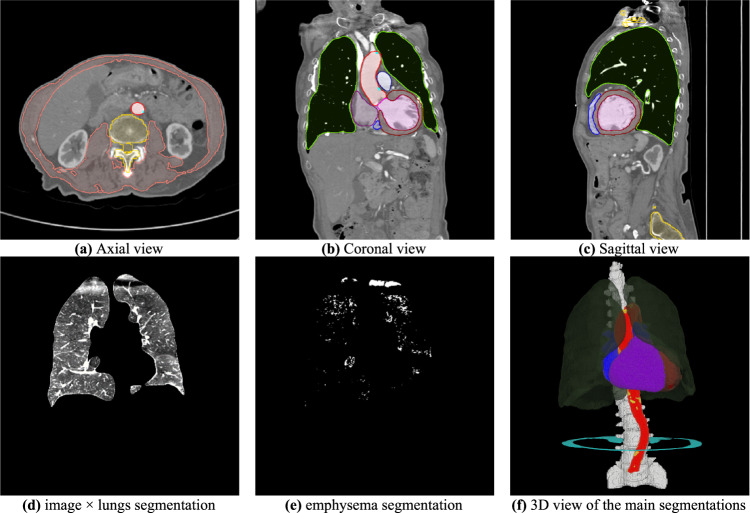
Illustrations of the different segmentations used in this study. **e** Is obtained from **d** using a threshold at −950 HU. **a**–**c** have a window set to [−400, 600] HU, while **d** is set to [−1000, −600] HU, to highlight the lungs’ structures. **f** illustrates the segmentations in 3D perspective (ParaView 5.12)

Segmentations of the heart chambers, great vessels, lungs, spine, and abdominal muscles at the L3 level were performed, with models previously described elsewhere [20, 21, 27], and illustrated in Fig. 1. TAC was then segmented using the previously described method. All used segmentation models were trained exclusively on their respective training data and were locked prior to their use on the TAVI-Lyon dataset.

The volumes of structures, such as heart chambers, lungs, and TAC, are directly derived from the segmentation. Abdominal belt muscles are calculated in the same manner, in 2D geometry. The L3 vertebra is located using the spine segmentation. Emphysema quantification is computed by collecting the percentage of voxels below a threshold of −950 HU [28–30] in the lungs. Finally, large vessel diameters are calculated using a topological thinning algorithm [31–33] applied to the segmentations.

All biomarkers except for emphysema ratio are provided in two sets: a standard measure, and a second one indexed to body surface area (BSAi), calculated according to the Boyd formula [34]. These biomarkers were calculated for all patients, and their statistics are reported in Table 2.

#### Selection of biomarkers and eCAPRI score development

Our methodology for variable selection and score calculation follows the one developed by Lantelme et al [14], using packages lifelines 0.27.8 and sklearn 1.3.0. During the initial univariate regressions, biomarkers are eliminated from the possible candidates if their *p*-value resulting from the likelihood ratio test is greater than 0.2. The high threshold on the *p*-value allows us to take into account possible confounding interaction factors [35].

While studying variable correlation, additional biomarkers are excluded if they reach a correlation higher than 0.85 with a CAPRI parameter or another biomarker that reaches a better *p*-value on the initial univariate regression.

Finally, as a third selection step, all combinations of the selected biomarkers were evaluated on the training set using a 4-fold cross-validation (75% training, 25% test). The tested biomarker combination is added to the original CAPRI score variables, and this augmented score is computed using the same methodology as the original. The augmented score showing the best mean AUC over the 4 folds is defined as the eCAPRI score, and the corresponding added biomarkers are set for the rest of the experiment. Once the variables of the eCAPRI score were set, the score was first fitted on the whole training set (without folds). The regression coefficients β and the mean values of the training set are retained to apply the score calculation on the evaluation set.

#### Evaluation

To evaluate the performance of this score, it was compared with the original CAPRI score, reweighted on our training set, and the EuroSCORE Logistic. The comparative 1-year death prediction ROC curves of the three scores are provided, along with their respective AUCs and 95% confidence intervals. A 10^4^-sample permutation test (bootstrap) is then calculated between the CAPRI and eCAPRI scores, and the resulting *p*-value is provided with the ROC curve. We also assessed the calibration of the CAPRI, eCAPRI, and EuroSCORE Logistic models to better evaluate their clinical applicability. The final coefficients β of both the CAPRI and eCAPRI scores fitted on the whole training dataset will be provided for further evaluation.

## Results

### Automated aortic calcium segmentation

**Fig. 2:**
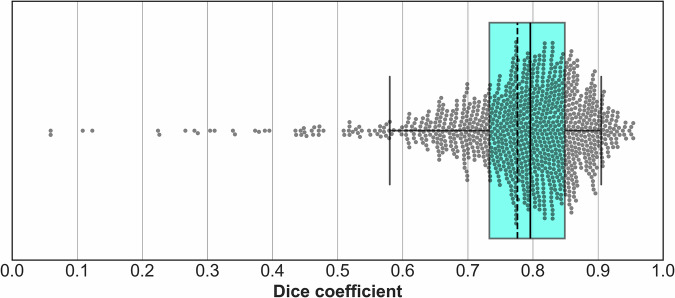
Distribution of Dice coefficients between predictions and ground truths of the aortic calcium segmentation. The box extends from the first to the third quartile, and the whiskers from the 5th to the 95th percentile. The median/mean is represented by a solid/dotted line in the box’s center

**Fig. 3:**
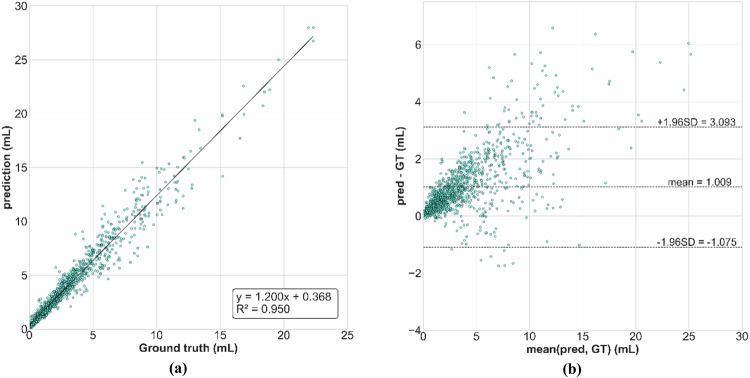
Comparison of the volume distributions of calcium predicted by the network and manually annotated using a scatter plot (**a**) and a Bland–Altman plot (**b**). GT and pred respectively stand for ground truth and prediction

**Fig. 4:**
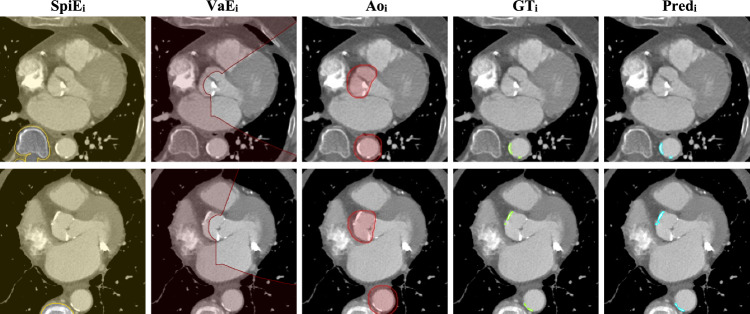
Examples of automated network segmentations (cyan—Pred_i_) compared to the corresponding ground truths (green—GT_i_). Valves (dark red—VaE_i_) and spine (yellow—SpiE_i_) exclusion masks and dilated aorta (red—Ao_i_) segmentation are also provided. Images are cropped around the heart to highlight the segmentation. A window of [−200, 1000] HU is applied to the images

The distribution of Dice coefficients resulting from the experiment is shown as a box plot in Fig. 2. The scatter plot and Bland–Altman plot of calcification volumes are grouped in Fig. 3, and some illustrations of the masks, ground truths, and predictions are gathered in Fig. 4.

The Dice coefficients range from 0.059 to 0.954 with an average of 0.777 ± 0.108. The 5th and 95th percentiles, first and third quartiles, and the median are 0.584, 0.905, 0.733, 0.849, and 0.796, respectively.

Regarding outliers, 60 of the 1111 patients in the evaluation set reached a Dice score inferior to 0.6, including 29 that are inferior to 0.5. The Dice coefficient is a demanding metric for this application, since it is highly sensitive to variations in small structures. The lowest Dice results presented here are due to scans with very low TAC volume (mean volume of 0.4 mL for the 29 patients with Dice inferior to 0.5, compared to 4.2 mL for the whole dataset*)*. Due to these low TAC volumes, the slightest disagreement between predictions and ground truths severely impacts the Dice score.

Amongst common segmentation errors, the network tends to include portions of the superior vena cava or adjacent artefacts when it is heavily injected and close enough to the aorta to be included in the dilated aorta mask. This limitation mainly affected contrast-enhanced scans in our dataset. Tracheal calcifications were sometimes included in the segmentation due to their proximity to the aorta. Finally, Fig. 3a shows the correspondence between GT and prediction. The slope of the linear fit indicates that predicted calcium volumes were slightly overestimated compared to the ground truth. This is illustrated in Fig. 4, in which the predictions appear thicker than their GT counterparts.

### The eCAPRI score

#### Biomarker selection

Regarding the extraction of biomarkers, their statistics are described in Table 2. Little variability is noticeable between the values of the biomarkers measured on the training and evaluation sets. The automatic segmentation methods occasionally failed, as evidenced by the minimum values of muscle surface area at L3 (2.44 cm²), or those of the maximum radii of the aorta and pulmonary arteries (smaller values of the order of a tenth of a millimeter) in a few CT scans.

The results of the univariate Cox regressions and ROC curves of the different biomarkers studied are detailed in Table 3. Pulmonary biomarkers appear to be largely uncorrelated with the risk of all-cause death at one year. Selected biomarkers are RA and RV volumes, aorta and pulmonary arteries max radii, abdominal belt muscles’ surface at L3 level, and all their BSA-indexed equivalent, along with BSA-indexed LV volume.

**Table 3:**
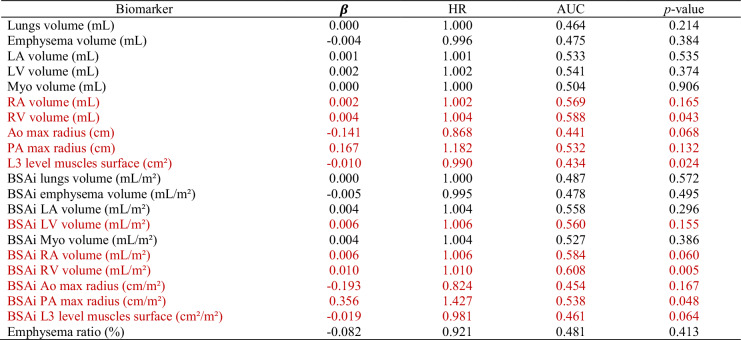
Results of the biomarkers’ univariate regressions for all-cause mortality at 1 year following TAVI implantation

Red rows indicate the selected biomarkers

*LA* left atrium, *LV* left ventricle, *Myo* myocardium, *RA* right atrium, *RV* right ventricle, *Ao* aorta, *PA* pulmonary arteries, *BSAi* body-surface-area-indexed, *β* regression coefficients, *HR* hazard ratios

**Fig. 5:**
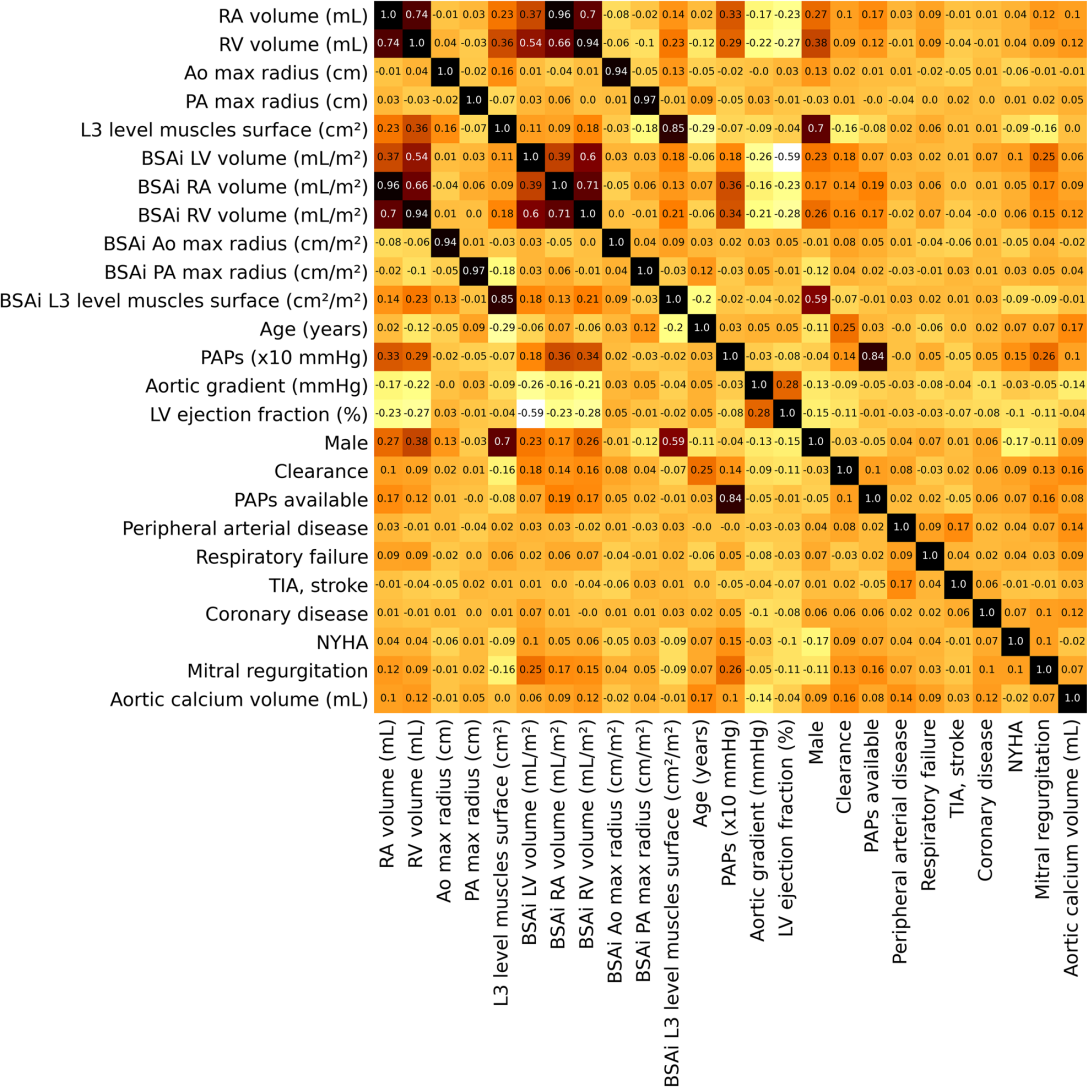
Correlation between retained biomarkers following univariate regressions and CAPRI score variables

The correlation coefficients gathered in Fig. 5 show significant correlations between the indexed and non-indexed biomarkers selected, as expected. In most cases, BSA-indexed measures outperformed their non-indexed counterparts (RA, RV, LV volumes, and PA max radius) during the univariate Cox regression and were selected. However, some biomarkers show better *p*-values when not indexed to the BSA (Ao max radius, L3 level muscles surface), which led to the elimination of said indexed measures. A strong correlation between the PAPs and PAPs availability score are also noted, which is intended, as a non-available PAP (availability = 0) is set to 0. The two variables are kept, to comply with the original CAPRI score methodology.

Finally, all biomarker combinations were tested during the 4-fold validation process of the score. The most optimized combination used the following biomarkers: BSA-indexed RV volume, BSA-indexed PA max diameter, a nd abdominal muscles surface at the L3 level. In the following validation study, the eCAPRI score was therefore calculated using the original CAPRI score variables and these biomarkers. Although the selection was data-driven, it is noteworthy that these three biomarkers are also clinically recognized as markers of right heart dysfunction, pulmonary hypertension, and frailty, respectively, which further supports their inclusion (see Discussion for details).

#### eCAPRI score evaluation

**Fig. 6:**
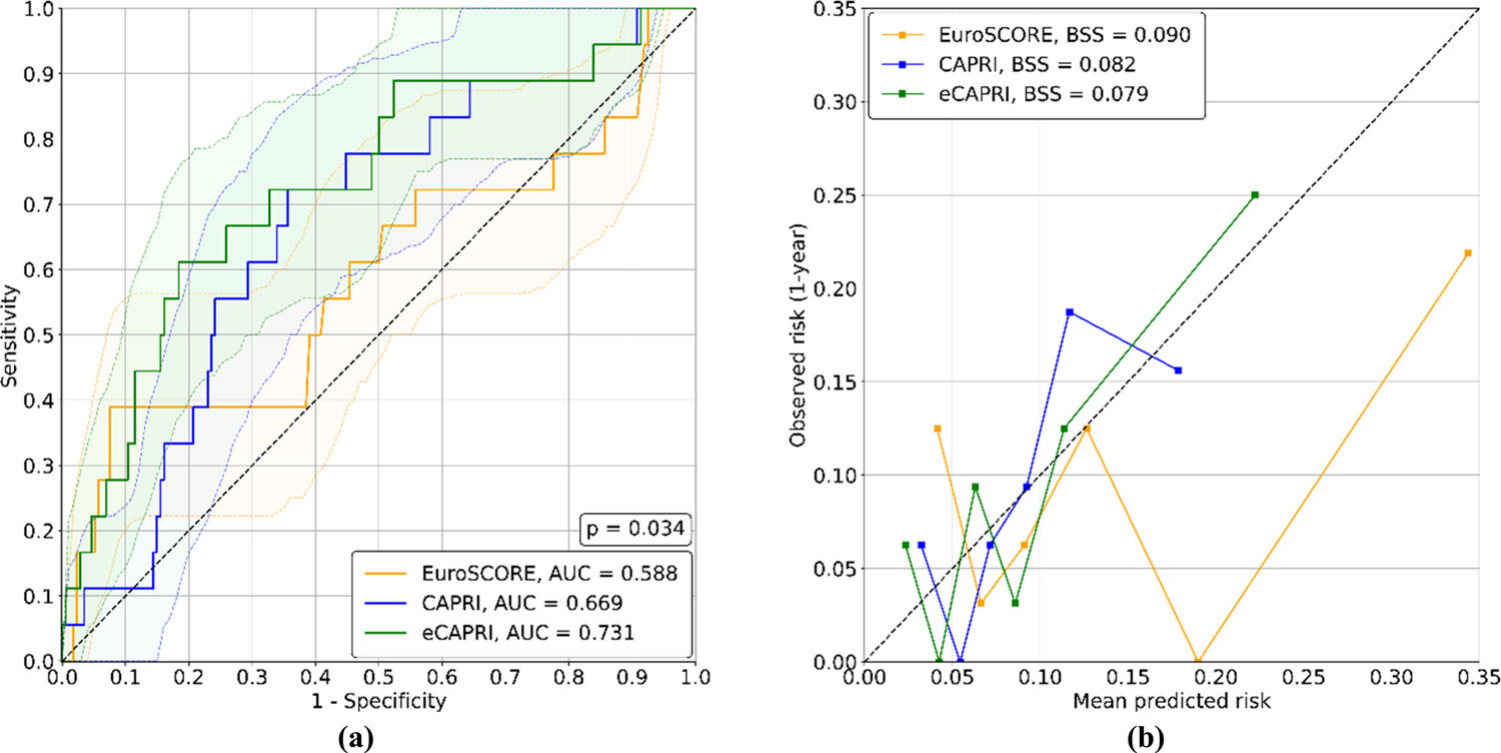
Compared the results of EuroSCORE, CAPRI, and eCAPRI for one-year all-cause mortality prediction. ROC curves (**a**) are displayed with 95% confidence ranges of corresponding color shades. The *p*-value calculated by a 10^4^-sample permutation test between the ROC curves of the CAPRI and eCAPRI scores is annotated on the graph. Calibration plots (**b**) of each method are also provided, with Brier skill score (BSS) annotated in the legend

Final coefficients of CAPRI and eCAPRI fitted on the whole training dataset are provided in Table 4. The EuroSCORE, CAPRI, and eCAPRI scores yield the results shown in Fig. 6 for the prediction of all-cause mortality at 1 year on the evaluation dataset. The AUC of the CAPRI and eCAPRI scores are significantly higher than that of the EuroSCORE (AUC = 0.588), traditionally used in clinical practice to assess surgical risk [36, 37]. Moreover, a significant difference (*p* < 0.04) can also be observed between the CAPRI and eCAPRI scores, which reach an AUC of 0.669 and 0.731, respectively.

**Table 4:** Final β Cox multivariate correlation coefficients of CAPRI and eCAPRI scores fitted on the whole training dataset and ${\bar{{{\rm{X}}}}}_{1}$ values of each variable

Biomarker	CAPRI β_i_	eCAPRI β_i_	$\bar{{{\rm{X}}}{{\rm{i}}}}$
Age (years)	0.0115	−0.0017	82.19
PAPs (×10 mmHg)	0.1620	0.1496	3.10
Aortic gradient (mmHg)	−0.0032	−0.0033	48.62
LV ejection fraction (%)	0.0027	0.0088	56.70
Male	0.1806	0.9495	0.51
Clearance	0.4433	0.3635	1.67
PAPs available	−0.3357	−0.3648	0.74
Peripheral arterial disease	0.0262	0.1262	0.12
Respiratory failure	0.1801	0.1569	0.20
TIA, stroke	0.3529	0.4121	0.11
Coronary disease	−0.4413	−0.4802	0.32
NYHA	0.1998	0.2177	1.41
Mitral regurgitation	0.0382	−0.0584	0.83
Aortic calcium volume (mL)	−0.0394	−0.0432	3.79
L3 level muscles surface (cm²)	-	−0.0221	120.32
BSAi RV volume (mL/m²)	-	0.0068	74.02
BSAi PA max radius (cm/m²)	-	0.3040	0.95

Calibration plots and Brier scores for the EuroSCORE Logistic, CAPRI, and eCAPRI scores are shown in Fig. 6b. The eCAPRI score demonstrated good calibration, with the lowest Brier score among the three models (0.090 for EuroSCORE Logistic, 0.082 for CAPRI, and 0.079 for eCAPRI, respectively), and a calibration curve closest to perfect calibration. The calibration plots for both CAPRI and EuroSCORE deviate from ideal calibration, with EuroSCORE notably underestimating risk in higher deciles.

## Discussion

In the present study, we managed to develop an updated version of the CAPRI score developed by Lantelme et al [14] for 1-year survival prediction after TAVI, providing faster computation and better predictive capabilities.

To simplify score computation, we implemented an efficient TAC segmentation method that rules out candidates outside the region of interest. This allows us to reach convincing results of a large dataset of 1111 contrasted thoracic scans, using a limited training dataset of 66 patients. Nonetheless, the network’s automated segmentation shows some limitations, when confronted with hardening artefacts near the aorta, or tracheal calcifications, which we cannot exclude from the candidates yet. Furthermore, the predictions tend to slightly overestimate the TAC volume. We then demonstrated that automatic segmentation of calcifications can be used to calculate the CAPRI score, which shows results similar to the original publication [14] (AUC = 0.669 and AUC = 0.680, respectively).

We then sought to further improve the CAPRI score using additional biomarkers directly extracted from the CT images. The choice to keep the methodology of the CAPRI score allowed us to establish the contribution of biomarkers to survival prediction, by comparing it to the original score. Biomarker selection was performed in three steps:

- Selection of correlated biomarkers using univariate analysis with a threshold on the resulting *p*-values of the likelihood ratio test.
- Exclusion of redundant biomarkers, assessed using correlation coefficients.
- Optimization of the best biomarkers’ combination using a 4-fold cross-validation on the training set.

Among the candidate biomarkers evaluated, BSA-indexed right ventricular (RV) volume, pulmonary artery (PA) maximal diameter, and L3-level muscle surface area were ultimately retained in the eCAPRI score. These three variables were selected not solely based on *p*-values from univariate Cox regressions, but because their inclusion yielded the greatest improvement in AUC during the 4-fold cross-validation procedure. Other variables with borderline significance (*p* < 0.2), such as BSA-indexed left ventricular volume or emphysema ratio, did not consistently improve model performance when included, or showed multicollinearity with already selected parameters. It is important to note that the threshold used to quantify the emphysema ratio (−950 HU) is debated in the literature [38], as iodine contrast injection may alter the ideal threshold value. This might account for the absence of the emphysema ratio from the selected biomarkers. Still, the biomarker selection is also supported by clinical rationale and literature:

- RV volume reflects right-sided cardiac remodeling, frequently associated with post-TAVI mortality, particularly in patients with pulmonary hypertension or right heart failure. Schmid et al [39] demonstrated an association between right ventricular volume and all-cause mortality at one-year post-TAVI, which seems to be confirmed by our results. They also note that LV functional parameters are of lesser influence on survival, which is also in line with our study.
- PA maximal diameter serves as an indirect marker of pulmonary vascular resistance and correlates with worse outcomes in valvular heart disease. It also appears to confirm the importance of the right circulation parameters for TAVI survival [40] and is associated with a higher risk of death than right ventricular volume in our study (HR = 1.427 and HR = 1.010, respectively).
- L3 muscle surface area is a surrogate of sarcopenia and frailty, both strong predictors of adverse outcomes in elderly TAVI populations [25, 27, 28].

These biomarkers were also less sensitive to imaging protocol variations and contrast timing compared to others (e.g., liver density, visceral fat ratio), ensuring greater robustness across centers.

The eCAPRI score that we developed in this study shows superior predictive capabilities (AUC = 0.731) to the original score (AUC = 0.669) and to the EuroSCORE (AUC = 0.588) classically used in clinical practice. Though not evaluated on the same dataset, it also shows convincing results compared to other papers, such as Agasthi et al [8] (AUC = 0.72), Penso et al [10] (AUC = 0.79 for 5-year outcome prediction). The calibration plots and Brier scores also indicate a better overall calibration and predictive accuracy of the eCAPRI score.

From a clinical perspective, the eCAPRI score has the potential to be seamlessly integrated into existing TAVI workflows. Since all TAVI candidates already undergo pre-procedural contrast-enhanced CT imaging, the required inputs for the score—including TAC volume and the additional imaging biomarkers—are already available in routine care. With the automation of segmentation and biomarker extraction, eCAPRI could be implemented as a background tool within radiological workstations or hospital PACS systems, providing clinicians with a standardized risk estimate without additional workload. This could support multidisciplinary medical teams in selecting patients who are likely to benefit from TAVI vs those for whom the procedure may be futile.

However, our study has some limitations. Though the eCAPRI score was developed and validated on a cohort including a broad spectrum of TAVI patients, ranging from moderate to high-risk individuals with diverse comorbidity profiles, our dataset is exclusively European, which may not fully represent anatomical or clinical variability seen in more diverse populations. In particular, the contributions to risk assessment of imaging biomarkers such as thoracic aortic calcification, muscle mass at the L3 level, and BSA-indexed cardiac dimensions may be altered across ethnicities due to differences in body composition and cardiovascular risk profiles. As such, the generalizability of the eCAPRI score to non-European populations remains to be established. To address this, we are currently in discussion for multiple international collaborations to furtherly validate our method on external data, from Switzerland and the USA. We will also get additional validation data from local centers in the following year. Such efforts will be crucial to assess the robustness and transportability of eCAPRI. The eCAPRI score should also be validated on a dataset that includes low-risk patients. Additionally, since the TAVI-Lyon dataset covers a large period of time from 2013 to 2021, future work should examine how evolving devices and techniques may have influenced TAVI outcomes over the study period. Finally, numerous TAVI survival scoring systems beyond CAPRI have been proposed in the literature. We aim to compare our approach with other scoring methodologies using a common dataset and explore the potential contribution of alternative predictors, including deep learning-based methods.

## Conclusion

The eCAPRI score combines fully automated extraction of imaging biomarkers with established clinical predictors to assess one-year mortality risk after TAVI. By eliminating the need for manual TAC volume annotation and incorporating additional imaging-derived features such as RV volume, PA diameter, and L3 muscle area, eCAPRI enables a standardized, reproducible, and time-efficient risk stratification process. Importantly, this approach provides clinicians with a tool that can support personalized decision-making by identifying patients at higher risk of adverse outcomes, thereby helping to avoid futile procedures and improve patient selection. While further investigations are nonetheless needed to confirm its performance on wider populations and comparison to other scores developed in the literature [8–10, 13], our proposed approach represents a meaningful step toward integrating advanced imaging analytics into routine pre-TAVI evaluation.

## Abbreviations

BSA: Body surface area
eCAPRI: Enhanced CAPRI score
TAC: Thoracic aortic calcium
TAVI: Transcatheter aortic valve implantation
